# Mapping Adult Vaccine Confidence in Future Health Professionals: A Pilot Study among Undergraduate Students at Two Universities in Greece

**DOI:** 10.3390/vaccines12070778

**Published:** 2024-07-15

**Authors:** Enada Leshi, Ilias Pagkozidis, Maria Exidari, Georgia Gioula, Maria Chatzidimitriou, Ilias Tirodimos, Theodoros Dardavesis, Zoi Tsimtsiou

**Affiliations:** 1 Department of Hygiene, Social-Preventive Medicine and Medical Statistics, School of Medicine, Aristotle University of Thessaloniki, 54124 Thessaloniki, Greece; enadaleshi@gmail.com (E.L.); pagkoilias@auth.gr (I.P.); ityrodim@auth.gr (I.T.); dardaves@auth.gr (T.D.); 2 Department of Microbiology, School of Medicine, Aristotle University of Thessaloniki, 54124 Thessaloniki, Greece; mexidari@auth.gr (M.E.); ggioula@auth.gr (G.G.); 3 Department of Biomedical Sciences, School of Health Sciences, International Hellenic University, 57400 Thessaloniki, Greece; chdimitr@ihu.gr

**Keywords:** adult vaccination, vaccine confidence, vaccine hesitancy, predictors, undergraduate, attitudes, Greece

## Abstract

Health professionals’ recommendations increase vaccine uptake. We aimed to document stances, practices regarding adult vaccination, and their predictors among undergraduate medical and biomedical science students, as well as their perspectives on increasing vaccine confidence. Among the 430 participants, third-year students from two universities in Greece, only 25.4% were in favor of all vaccines, while no refuters were detected. Predictors of recommending vaccination were the Attitudes Towards Adult Vaccination (ATAVAC) Value (OR 3.26, *p* < 0.001) and ATAVAC Safety subscales scores (OR 1.36, *p* < 0.05), being a medical student (OR 2.45, *p* < 0.05), and having better self-rated health status (OR 2.27, *p* < 0.05). The importance of getting vaccinated as health professionals was recognized by participants with a higher ATAVAC value (OR 5.39, *p* < 0.001), ATAVAC Safety scores (OR 1.46, *p* < 0.05), and increased knowledge regarding the National Immunization Program (OR 1.31, *p* < 0.05). The God Locus of Health Control (GLHC) was a predictor only in vaccination against COVID-19 (OR 0.91, *p* < 0.05). Improving community health literacy and health providers’ education, boosting trust in authorities, and adopting a person-centered approach emerged as the main themes regarding how to increase vaccine confidence. Mapping health professionals’ confidence in vaccines and providing lifelong training support is pivotal in supporting positive attitudes, enhancing their competence, and promoting vaccination in the post-COVID-19 era.

## 1. Introduction

Vaccination constitutes one of the most successful and cost-effective public health interventions [1,2], currently estimated to prevent around three million deaths worldwide [3]. An integral arsenal of preventive medicine and public health, vaccines contribute to reducing the burden of infectious diseases, eradicating others, and improving the quality of life [4,5]. Increasing vaccination coverage is of paramount importance as 1.5 million deaths could be additionally averted [3] and healthcare costs attributed to vaccine-preventable diseases skyrocket [1]. Health professionals’ endorsement and recommendation is key in boosting vaccine uptake [3,6,7,8].

Vaccination coverage in the adult population is reportedly poor, a finding often attributed to immunization-related hesitancy. The World Health Organization considers vaccine hesitancy, i.e., the postponement, skepticism or refusal of immunization despite the availability of such services, to be one of the ten threats to public health [3,9,10]. Individuals’ stances towards immunization are represented on a continuum ranging from the following responses: acceptance of all vaccinations to various degrees of hesitancy; agreement with concerns; acceptance of some, delay or refusal of others; unwillingness regarding immunization, yet having doubts; and refusal of all vaccines with conviction [10,11]. Immunization-related hesitancy is a behavioral phenomenon that fluctuates according to a given context, time, place, and type of vaccine [9,10]. As such, it is not uncommon even for those vaccinated in the past to develop significant doubts and concerns regarding vaccines, thus impeding future immunizations [12,13]. The framework of “5Cs”, i.e., mistrust in vaccine safety, efficacy, value, and the authorities (“confidence”), low susceptibility to disease (“complacency”), negligence, availability, affordability, and accessibility of immunization programs (“convenience”), absence of recommendation, poor awareness, insufficient information, and evidence synthesis (“calculation”) and unwillingness to protect others (“collective responsibility”), has been proposed to explain vaccine hesitancy [14]. During the COVID-19 pandemic, mistrust in the authorities, the development process, vaccine safety and effectiveness, low risk perception, and missteps in public health communication resulted in an upsurge in hesitancy [15]. Religious beliefs and notions that a higher power is in control of individual’s health was reported to hamper adherence to restrictions during the pandemic as well as uptake of the novel COVID-19 vaccines [16]. In Greece, hesitancy among health professionals became evident, once the authorities established mandatory COVID-19 vaccination for health workers [17].

Health professionals’ stances, beliefs and advice essentially affect the public’s trust in the immunization process and vaccine acceptance [3,6,7,8]. They are trusted consultants, shape patients’ viewpoints, and influence their decisions regarding vaccination, battling the ever-emerging hesitancy [3,18]. Acting as role models within their respective communities, they promote vaccinations and aid in the successful implementation of public health policies. The literature has long highlighted the correlation between health providers’ affirmative stances and the increased uptake and coverage among the population they are responsible for [19]. They are viewed as primary, reliable sources of information and their advice and recommendation does reinforce vaccination uptake [20,21,22]. Indeed, recent studies underscore that patients protected against vaccine-preventable diseases are up to 74 times more likely to have been recommended vaccination by their doctor [23,24]. With regard to inoculating health professionals, the role of university curricula in the development of immunization-related affirmative professional stances and behaviors among health science students is underscored [25]. Attitudes and practices at training and early professional work stages are expected to portray future preparedness and competence as practicing health providers.

Research on health science students’ stances across the vaccine acceptance continuum, as well as on their views on how to increase vaccine confidence, is lacking. There are a few studies that have documented stances and practices among undergraduate students of healthcare professions, specifically in medical [26] and dental [27] students, during the pandemic. This study is, to the best of the authors’ knowledge, the first to document stances, practices, and predictors thereof, as well as perspectives on boosting adult vaccine confidence among undergraduate medical and biomedical science students in the aftermath of the pandemic. Moreover, it is also the first to explore the connection of vaccination attitudes and practices and the notion among future health professionals that God is in control of one’s health status. Exploring future health professionals’ perceptions and practices towards adult vaccination and determinants thereof are integral in designing effective vaccination programs as well as interventions to overcome barriers in training and competence [26], whilst empowering future health professionals to confidently address immunization-related hesitancy and enhance vaccine promotion in the post-pandemic era.

## 2. Materials and Methods

### 2.1. Study Design and Setting

A pilot study was conducted to map confidence, stances, and practices regarding adult vaccination among undergraduate medical and biomedical science students, as well as their perspectives on how to increase vaccine confidence. Data were collected from third-year students enrolled in the School of Medicine, Aristotle University of Thessaloniki (AUTh), the largest University in Greece, and the Department of Biomedical Sciences, International Hellenic University (IHU), also situated in the same city as the former. Students from across the country register in both universities, following success in national entrance exams. Through the undergraduate curricula, biomedical science students train and delve into the execution, causes of variability, and assurance of laboratory tests. Upon graduation, they primarily equip diagnostic centers, executing and assuring laboratory tests. To a lesser degree than their medical counterparts, biomedical science graduates have patient-facing responsibilities, encountering patients during registration, blood drawing, and verification and handover of results. Biomedical scientists are healthcare professionals that are expected to form relationships of trust and care with the community, act as points-of-contact with the health system, and may offer opportunistic health-related advice, whereas medical graduates primarily care for and counsel patients.

### 2.2. Study Population

All third-year undergraduate students at the School of Medicine, Faculty of Health Sciences, AUTh and the Department of Biomedical Sciences, School of Health Sciences, IHU were invited to take part in the study. As no published data regarding health sciences students’ standpoint in the continuum of vaccine acceptance were publicly available at the time of study design, no sample size estimate was performed. The research team strived, instead, to recruit the highest rate of the target population in this pilot study. In total, 297 and 150 third-year students were enrolled in the School of Medicine and the Department of Biomedical Sciences, respectively, in the academic year 2022–2023. The study ran in March 2023, during the spring semester.

Students had the opportunity to attend vaccination-related lectures, which were held during the previous semester of the same academic year for both schools. Medical students had attended two two-hour classes on the National Immunization Programs and the need to increase vaccine confidence and coverage, while biomedical science students had attended one similar one-hour class. Third-year medical and biomedical science students were selected as they commence clinical rotations during the spring semester of this academic year and therefore underscoring their knowledge and confidence related to adult vaccination was considered important.

Participants were informed and invited to participate in the study during compulsory classes to ensure the approach of all third-year students. Each student group attending those classes was approached once for recruitment purposes. Participation was anonymous and optional. Completion of the study tool indicated consent. To minimize social desirability bias, hard copies of the study tool were returned in an opaque ballot box at the course site. Students were provided no incentives to take part in the study. It should be reported that teaching professors in both schools explained the aim of the study at the end of the class and encouraged students’ anonymous and voluntarily participation.

### 2.3. Study Tool

The study tool consisted of 39 questions/items in four distinct parts and required approximately nine minutes to complete. Participants first completed a sociodemographic survey, documenting the age, gender, department of attendance, history of diagnosis of a chronic disease, smoking habit, and self-rated health status (on a 5-point Likert scale, ranging from 1 = excellent to 5 = poor).

The second part of the study tool explored participants’ attitudes with regard to adult vaccination, utilizing the Attitude Towards Adult Vaccination (ATAVAC) scale [27]. This is a valid and reliable scale, developed in the Greek language, comprising of 11 items, in which participants respond with their degree of agreement using a 6-point Likert scale (ranging from 1 = strongly agree to 6 = strongly disagree). The scale consists of three subscales: (a) “Value of adult vaccination” (7 items); (b) “Safety concerns” (2 items); and (c) “Perceived barriers” (2 items). Participant’s scores range from 1 to 6, with higher ATAVAC scores signifying favorable attitudes towards adult immunization. Higher subscale scores indicate positive viewpoints regarding the value of adult vaccination (ATAVAC Value of adult vaccination), fewer concerns over the vaccine’s safety profile (ATAVAC Safety concerns) and fewer barriers towards vaccination (ATAVAC Perceived barriers). Participants were asked to respond, utilizing the same 6-point Likert scale, with their degree of agreement with three additional items, inquiring about fear of needles, negative experiences with vaccinations in acquaintances, and beliefs about pharmaceutical companies’ profits and vaccines.

In the third part, we explored participants’ perceptions and practices regarding adult vaccination. Students were asked to identify themselves across the five categories of the vaccine acceptance continuum, as follows: (a) accept all adult vaccines; (b) accept adult vaccines, but unsure about all; (c) accept some adult vaccines, but delay or refuse some; (d) refuse adult vaccines, but unsure about all; and (e) refuse all adult vaccines [10]. Additional data were collected utilizing a 10-point Likert scale on the following: (a) the importance of getting vaccinated as health science students and health professionals (ranging from 1 = not important at all, to 10 = extremely important); (b) their self-reported level of information with regard to motivating others to vaccinate according to national guidelines (ranging from 1 = not at all, to 10 = excellent level of information); (c) their self-reported confidence to address vaccine hesitancy in patient encounters (ranging from 1 = not at all, to 10 = extremely confident). Participants’ stances on vaccination endorsement in future patient encounters (dichotomous answer: Yes/No) as well as their self-reported personal vaccination status were documented. Data on the history of the students’ vaccinations consisted of the COVID-19 vaccine, as well as recommended vaccinations according to the 2023 National Adult Immunization Program against seasonal influenza (health science students are recommended one dose each season as a high-risk group), tetanus, diphtheria, pertussis (one booster dose for those 18–25 years old followed by a booster dose every 10 years) and human papillomavirus (HPV). With regard to the latter, participants were asked to indicate their status according to vaccination history in childhood, adolescence (universal vaccination for those aged 9–18 years old) or adulthood (vaccination for those 18–26 years old belonging in high-risk groups), according to the 2023 Childhood and Adolescence [28], and Adult Immunization Programs [29]. Ultimately, an open-ended question explored participants’ suggestions on facilitators in addressing vaccine hesitancy as inoculating health professionals.

The fourth part consisted of the validated Greek version of the God Locus of Health Control (GLHC) scale, reflecting participants’ belief in attributing control of their health conditions and status to God [30,31]. Participants responded to 6 items using a 6-point Likert scale (ranging from 1 = strongly disagree to 6 = strongly agree). Scores ranged from 6 to 36, with the highest ones indicating a greater attribution of health control to God. 

The study tool was pre-tested to assess the level of comprehension in a convenience sample of 10 undergraduate students prior to study commencement. Five third-year biomedical science and five medical students completed the study tool and took part in debriefing interviews that indicated good understanding of the study tool. The data from these 10 completed study tools were not included in the analysis.

### 2.4. Data Analysis

Since the proportion of missing values per item/question was below 5% (ranging from 0.7–1.9%), no items were excluded from the analysis, while pairwise deletion was used to handle missing values [32]. To examine the normality of continuous variables, the Shapiro–Wilk test was performed. Mean (M) and standard deviation (SD) summarize normally distributed variables, the median and interquartile range (IQR: Q1, Q3) summarize non-parametric variables, whereas categorical ones are presented as absolute and relative frequencies among responders—n/N (%). Associations between non-normally distributed scores were examined using Mann–Whitney U tests, whereas correlation between continuous variables was examined by Spearman’s correlation coefficient.

To investigate potential prognosticators of participants’ beliefs and uptake of adult vaccination, logistic regression analyses were performed. Outcome variables were as follows: (a) the importance of getting vaccinated as health sciences students (≥9 on a 10-point Likert scale was set as the cut-off point indicating importance); (b) the importance of getting vaccinated as health professionals (≥9 on a 10-point Likert scale was set as the cut-off point indicating importance); (c) endorsement of vaccination in future patient encounters; (d) being vaccinated as an adult; and (e) uptake of COVID-19, influenza, tetanus, diphtheria, pertussis, and HPV vaccines. For each outcome, univariable analyses were performed fitting only one independent variable. Explanatory variables were as follows: (a) sociodemographic variables such as gender and study department/school; (b) health status variables such as self-reported health assessment (excellent vs. other), presence of a chronic condition, and adult vaccination history; (c) vaccination-related beliefs and attitudes such as accepting all adult vaccines, being informed about vaccination guidelines, being confident to manage vaccine hesitancy (≥9 on a 10-point Likert scale was set as the cut-off point indicating confidence), having no fear of needles, and believing that vaccines are not a way for pharmaceutical companies to make money; (d) ATAVAC subscales’ and (e) GLHC scores. Explanatory variables with statistically significant outcomes in the univariable analyses were included in the final, multivariable models and are presented in the following tables. Adjusted odds ratios (aOR) are presented along with corresponding 95% confidence intervals (95% CI). The variance inflation factors (VIFs) of all the independent variables suggested no multi-collinearity (VIF range 1.01–1.78). The significance level was set at 0.05 and 2-tailed. Data were processed using Jamovi (Version 2.4, The jamovi project (2024), Sydney, Australia).

Study participants were invited to elaborate on their suggestions to increase vaccine confidence through an open-ended question in the study tool. Collected data were examined independently by two authors, one biomedical scientist and one physician, both trained and experienced in qualitative data analysis. The authors open coded the available data, and identified, agreed upon, applied, and iteratively refined a coding framework to all participant responses. Four key themes were identified through thematic content analysis, up to reaching saturation [33].

## 3. Results

In total, 430 individuals took part in the study (94.6% and 99.3% response rates in medical and biomedical science students, respectively). The sociodemographic characteristics of the participants, as well as information regarding their health status, are illustrated in Table 1. The median age of participants was 21 years (IQR: 20, 21, min. 20–max. 42). A known diagnosis for chronic disease was reported by 11.2% (*n* = 40) of study participants.

Regarding participants’ belief in God being the locus of their health or specific health-related condition control, mean GLHC scores for medical and biomedical science students were 11.6 (SD = 6.86, min. 6–max. 36) and 12.2 (SD = 7.01, min. 6–max. 36), respectively. The GLHC scale showcased excellent internal consistency (Cronbach’s alpha a = 0.98) in both medical and biomedical science students. Participants’ scores on the six items of the GLHC scale are depicted in Table 2.

### 3.1. Attitudes towards Adult Vaccination

Regarding participants’ standpoints within the vaccine acceptance continuum, 25.4% were in favor of all adult vaccines, whilst the rest underscored various degrees of hesitancy (‘in favor of adult vaccines, but not sure about all’: 65.3%, ‘in favor of some adult vaccines, but not sure about all’: 8.6%, ‘against adult vaccines, but not sure about all’: 0.7%), as illustrated in Table 3.

Medical students’ mean score on the ATAVAC scale was 5.11 (SD = 0.52, min. 3.36–max. 6). Their subscale scores were as follows: (a) Value of adult vaccination 5.24 (SD = 0.52, min. 3.14–max. 6); (b) Safety concerns 4.38 (SD = 1.20, min. 1–max. 6); and (c) perceived barriers 5.41 (SD = 0.65, min. 2.5–max. 6). Respective scores for participants in the Biomedical Science department were as follows: (a) ATAVAC 4.84 (SD = 0.55, min. 3.27–max. 5.91); (b) Value of adult vaccination 4.97 (SD = 0.57, min. 3.14–max. 6); (c) Safety concerns 4.02 (SD = 1.28, min. 1–max. 6); and (d) Perceived barriers 5.18 (SD = 0.71, min. 2.5–max. 6). ATAVAC’s internal consistency in medical and biomedical science students was satisfactory (Cronbach’s alpha a = 0.77 and a = 0.78, respectively). Total ATAVAC, ATAVAC subscale and GLHC scores pursuant to participants’ stances within the immunization acceptance spectrum are underscored in Table 4. Mean scores per ATAVAC item and perceptions on additional hesitancy-related inquiries are highlighted in Table 5. Negative, weak correlations were noted between GLHC and total ATAVAC (Spearman’s rho = −0.35, *p* < 0.001), ATAVAC Value of adult vaccination (Spearman’s rho = −0.29, *p* < 0.001), ATAVAC Safety concerns (Spearman’s rho = −0.35, *p* < 0.001), and ATAVAC Perceived barriers (Spearman’s rho = −0.132, *p* < 0.001) subscales.

The participants’ beliefs over health professionals’ roles in adult vaccination are highlighted in Table 6. The importance of adult vaccination amongst health sciences students as well as health professionals was more valued by medical students than their biomedical science counterparts; z = −4.628, *p* < 0.001 and z = −3.447, *p* < 0.001, respectively. The multivariable logistic regression revealed that the importance of getting vaccinated as health sciences students was emphasized by female participants (OR: 2.48; 95% CI: 1.40–4.39; *p* = 0.002), medical students (OR: 2.03; 95% CI: 1.18–3.47; *p* < 0.001), those with a high ATAVAC Value (OR: 3.43; 95% CI: 1.83–6.43; *p* < 0.001), ATAVAC Safety scores (OR: 1.38; 95% CI: 1.08–1.77; *p* = 0.011), and those better informed about vaccination guidelines (OR: 1.38; 95% CI: 1.15–1.65; *p* < 0.001) (Table 7). Students with increased ATAVAC Value (OR: 5.39; 95% CI: 2.55–11.40; *p* < 0.001), ATAVAC Safety scores (OR: 1.46; 95% CI: 1.07–11.98; *p* = 0.016), and knowledge regarding the National Immunization Program (OR: 1.31; 95% CI: 1.07–1.59; *p* = 0.008) were more likely to accentuate the importance of getting vaccinated as health professionals (Table 7). Participants from both departments felt equally informed to motivate close acquaintances to vaccinate themselves according to official guidelines (z = −1.778, *p* = 0.075), whilst also reporting similar rates of confidence in addressing vaccine hesitancy in the future (z = −1.067, *p* = 0.286). In total, 242 medical (86.7%) and 97 biomedical science students (65.1%) would urge future patients to get vaccinated. The multivariable logistic regression revealed that medical students (OR: 2.45; 95% CI: 1.34–4.48; *p* = 0.004), those with increased trust in the value of adult immunization (ATAVAC Value subscale score, OR: 3.26; 95% CI: 1.65–6.42; *p* < 0.001), and those with fewer concerns regarding vaccine safety (ATAVAC Safety subscale score, OR: 1.36; 95% CI: 1.02–1.80; *p* = 0.037), as well as participants with excellent self-rated health status (OR: 2.27; 95% CI: 1.14–4.52; *p* = 0.020) were more likely to urge their patients to get vaccinated according to the National Adult Vaccination Program guidelines (Table 7).

### 3.2. Predictors of Vaccine Uptake

The vast majority of medical (90.4%) and biomedical science students (85.8%) reported at least one vaccination as an adult. The self-reported vaccination history of participants is illustrated in Table 8. The multivariable logistic regression revealed that the predictors for vaccination uptake as an adult amongst participants were higher ATAVAC Value scores (OR: 2.18; 95% CI: 1.09–4.35; *p* = 0.027) and self-rated awareness of vaccination guidelines (OR: 1.19; 95% CI: 1.02–1.39; *p* = 0.032) (Table 9). The presence of a chronic health condition was the only predictor for influenza vaccine uptake (OR: 2.86; 95% CI: 1.46–5.58; *p* = 0.002) (Table 9). Being informed about the vaccination program (OR: 1.21; 95% CI: 1.06–1.39; *p* = 0.005) and scoring lower in the ATAVAC Value subscale (OR: 0.56; 95% CI: 0.36–0.88; *p* = 0.011) were the predictors for tetanus, diphtheria, and pertussis vaccine uptake (Table 9). Predictors for COVID-19 vaccine uptake were a high ATAVAC Value (OR: 4.80; 95% CI: 1.86–12.41; *p* = 0.001), ATAVAC Safety (OR: 1.71; 95% CI: 1.12–2.60; *p* = 0.013), low GLHC scores (OR: 0.91; 95% CI: 0.85–0.97; *p* = 0.003), and being a medical student (OR: 2.65; 95% CI: 1.06–6.61; *p* = 0.037) (Table 9). The multivariable logistic regression revealed that the predictors for HPV vaccine uptake were female gender (OR: 19.45; 95% CI: 10.06–37.63; *p* < 0.001), and increased trust in adult vaccination (ATAVAC Value subscale score, OR: 2.66; 95% CI: 1.41–5.01; *p* = 0.002) as well as fewer concerns over vaccine safety (ATAVAC Safety subscale score, OR: 1.48; 95% CI: 1.12–1.95; *p* = 0.006) (Table 9).

### 3.3. Mitigating Vaccine Hesitancy

Regarding enablers in addressing vaccine hesitancy, four main themes emerged from the analysis of participant’s responses to the open-ended question: (a) increased health literacy in the community; (b) a patient-centered approach towards users of health services; (c) improving health professionals’ training and education; and (d) boosting trust in authorities and institutions. Study participants primarily valued the importance of advancing health literacy in the community. Seminars, campaigns, and interventions, run by health professionals, educators, and local authorities alike, are key in increasing awareness, understanding the value, and strengthening confidence in vaccines. In future consultations, participants suggested adapting arguments according to patient’s characteristics and beliefs with regard to the acceptance continuum, utilizing comprehensible language and terms, fomenting discussion in the safe patient–provider space, and addressing misconceptions and stereotypical beliefs with evidence-based arguments. Participants recognized the importance of university and lifelong training to better equip inoculating health professionals with the skills to increase vaccine confidence and uptake. Expertise regarding immunization, which also extends to production and supply chain information and updated knowledge on recommended vaccination schedules, is key in addressing patient inquiries, whilst mitigating hesitancy in health providers. In a like manner, communication skills and hesitancy-related training enable students to effectively approach patients across the acceptance continuum. Ultimately, restoring and strengthening trust in institutions is of utmost importance, as participants underscored the need to increase transparency in vaccine production, the supply chain, and public health authorities. Enhancing accessibility and renouncing compulsory acts, whilst promoting the social responsibility of getting vaccinated, will reinforce vaccine confidence. The main themes along with selected illustrative quotes are presented in Table 10.

## 4. Discussion

### 4.1. Main Findings

In this study, we investigated attitudes and practices related to adult vaccination in undergraduate medical and biomedical science students in Greece. In general, participants from both departments recognized the value of adult vaccination, had few concerns over vaccine safety, and faced few barriers in getting vaccinated. Medical students’ scores on the ATAVAC Value and Safety subscales were significantly higher than their biomedical science counterparts. Yet nearly three fourths of participants self-reported various degrees of hesitancy across the vaccine acceptance continuum. Most future health professionals would encourage patients to get vaccinated according to national guidelines. Those believing in the value of vaccination, having fewer safety concerns, and excellent health status, as well as medical students, were more likely to urge such patients. To address vaccine hesitancy, participants called for increasing health literacy in the community and public trust in the authorities, providing educational opportunities in university and lifelong training for health providers, as well as adopting person-centered approaches to combat hesitancy in future consultations. Regarding vaccination practices, most students had had at least one vaccination as adults, primarily against COVID-19, with awareness of vaccination guidelines and trust in the value of vaccination being the related predictors. Vaccine uptake against tetanus, diphtheria, pertussis, and HPV was insufficient, whereas coverage against influenza was the lowest. Although participants’ God Locus of Health Control was found to be weakly negatively correlated with beliefs over the value of immunization and their concerns regarding vaccine safety, it was not among the predictors of getting vaccinated as students or health professionals or of suggesting vaccination in their future patients. This belief was a predictor for immunization solely in vaccination against COVID-19.

### 4.2. Comparison with the Existing Literature

One-fourth of the study participants were in favor of all adult vaccinations. Whereas the majority voiced various degrees of hesitancy towards immunization, participants in general leaned on the positive side of the vaccine acceptance continuum. It is worth noting that no vaccine refuters were noted among the study participants, a representative finding for third-year medical and biomedical science students given the study’s high response rates. Vaccine refuters also constituted a small proportion of medical students in Bosnia and Herzegovina and Croatia, where one-third and 15% were recorded as hesitant towards adult vaccination, respectively [34]. Those considering the benefit–risk ratio to be negative for all vaccines also constituted a fraction of participants in former studies among French health sciences students [35] and General Practitioners (GPs) [36]. As this is, to the best of our knowledge, the first effort to place students across the vaccine acceptance continuum, further research is needed to accordingly document hesitancy in this population.

Participants were confident regarding adult immunization, as reflected on their ATAVAC scale scores. The value of adult vaccination was highly regarded, whilst few concerns over vaccine safety and barriers to accessing vaccinations were underscored. Scores in both departments were concordant with participants’ stances within the continuum of vaccine acceptance, with those reporting hesitancy scoring lower in the total and respective subscales. Though not utilizing validated tools, a French study also demonstrated low hesitancy and increased perception of vaccine safety among health sciences students [35]. Low rates of hesitancy were also noted in American nursing, medical and pharmacy students [37]. Spanish nursing, physiotherapy and chiropody students scored on the positive end of a validated scale for health science students, highlighting favorable attitudes, behaviors and beliefs towards vaccination before the start of the COVID-19 pandemic [38]. Nursing students in Canada scored high in the Vaccine Acceptance Instrument indicating positive attitudes towards adult vaccines [39]. Positive attitudes towards vaccination were also noted among medical students in Serbia [40] and Nepal [41], whereas Saudi medical students’ attitudes were reportedly moderate [42].

Manifested through scores on the low end of the GLHC scale, participants in both departments did not consider God to be in control of their health status and conditions. The notion that God is the locus of control of children’s health and, therefore actions to avert vaccine-preventable diseases is a matter of His will, is reported among Dutch Orthodox Protestant parents who did not adopt the immunization program. For these parents, vaccination was reportedly accepted when imminent danger was in sight, as in the case of epidemics [43]. Belief in God’s ability to intervene is negatively associated with COVID-19 vaccine uptake in the US general population [16], a finding echoed in our study results. As research on the effect of the role of God in students’ health is scant, further research is needed to explore unearthed associations in different cultural settings.

Study participants considered adult vaccination to be important for health students, a belief also noted in Canadian nursing, pharmacy and medical students [6,44]. In a like manner, the ethical obligation to be vaccinated against influenza as health science students was noted in US [37], Australian [45] and Spanish studies [38,46]. Spanish students also noted the importance of being vaccinated so as to reduce transmission of infectious diseases during clinical rotations [38]. Moreover, our participants acknowledged immunization in adulthood to be important for those working as health professionals. This notion is echoed in Canadian [6], US [37], and Spanish counterparts, who report willingness to undergo all available vaccinations once they start working as health professionals [38]. Greek dental and Italian nursing students also consider vaccinations against preventable diseases to be important for practicing health care personnel [47,48]. Australian medical students also agree on the importance of health workers’ influenza vaccination being updated each year [45].

Health professionals’ advice and endorsement of vaccinations have long been reported as key drivers to increase vaccine confidence and uptake [6,7,8]. Increased recommendation rates were underscored among Italian nursing and French and Spanish health sciences students, with rates not being significantly different within departments, like their Greek counterparts [35,38,48]. Medical students in our study, those who value adult vaccination and have fewer concerns over safety, were more likely to urge their future patients to get vaccinated according to national guidelines. Finnish health providers alike recommend vaccination to their patients if they believe in the vaccine’s value and safety profiles [49]. Our finding is in line with those of previous studies demonstrating low confidence in vaccine safety and benefits as hindering health professionals from getting vaccinated themselves and recommending vaccination to patients [18,36,49,50,51,52].

Health science students in our study reported being moderately informed to motivate others to get vaccinated. In a similar manner, Croatian and Bosnian medical students self-report inadequate levels of information regarding adult vaccination [34]. Italian primary care pediatricians as well as US medical students also feel inadequately informed and underscore the need for more information regarding HPV infection and vaccines [52,53]. In addition, our participants felt moderately confident in addressing vaccine hesitancy in encounters with future patients. US pediatricians and Australian primary care providers also note difficulties in responding to hesitant patients’ inquiries [54,55], whereas French GPs report moderate confidence in explaining vaccine safety themes to patients [36]. Canadian health science and US and Nepalese medical students report feeling uncomfortable when discussing patients’ concerns about the safety profile of vaccines [6,41,56].

Vaccination rates among participants in our study were suboptimal. Greek students’ trust in the value of immunization and knowledge of vaccination guidelines were significant predictors in getting vaccinated. Whilst health science students’ belief in vaccine effectiveness and safety did not predict vaccination coverage in France, medical students were more likely to be vaccinated, whereas the department of origin did not impact the vaccination history in our participants [35]. With regard to individual vaccines, the high rates for COVID-19 vaccination reported in our study may be interpreted under the decision for mandatory vaccination in health science students, with failure to comply leading to exclusion from clinical rotations [57], hence the widespread coverage reported in Greek dental students (87.7%) as well [47]. Contrary to our findings, tetanus, diphtheria, and pertussis vaccine uptake is reportedly increased in French (94.8%), Greek health science (70.4%) [35,58] and Greek dental students (63.2%) [47]. Coverage rates for influenza were insufficiently reported in our study, a finding echoing those of previous studies in health professionals and health science students alike [36,38,47,58,59,60,61,62,63]. Yet coverage for influenza was reportedly widespread among Canadian health science students (85%) [6,44] and moderate among Australian medical students (53.8%) [45]. Though our study highlighted that attitudes towards adult vaccination do not significantly predict uptake of the influenza vaccine, Spanish health science students and Finnish health professionals with positive standpoints were more likely to get vaccinated [38,49]. Suffering from a chronic condition predicted vaccination against influenza in our study as well as in health care workers in Spain and Italy [64]. Regarding HPV vaccine uptake, French (61.8%) and Greek (75.6%) studies recorded comparable rates with our participants [35,58], whereas fewer health science students were covered against HPV in the US (44.1%) [53] and Italy; 40.5% in a study by Mascaro et al. and 23.9% in one by Pelullo et al. [65,66].

In combatting vaccine hesitancy and increasing confidence amongst health science students, four strategic aims were underscored by our participants. Appropriately informing communities about vaccine-preventable diseases, the way vaccines work, their benefits and risks, and details regarding the vaccination program allow for a common understanding of vaccination that is reportedly influencing individuals’ decisions on getting vaccinated [7]. Community outreach and utilizing evidence-based medicine is crucial in strengthening vaccine literacy, promoting acceptance, and battling misinformation circulated online and through mass media [7,51]. When communicating with hesitant patients, our participants suggested adopting a person-centered approach. Permission to engage in vaccine-related conversations, the use of content according to patient’s concerns, open-ended questions, and language corresponding to their health literacy level are suggestions also proposed by Australian primary care professionals [55]. Like international students [6,37,41,46,61], participants in our study noted insufficient training and called for in-depth analysis of vaccines and immunology in undergraduate courses, as well as the introduction of soft skills classes focusing on enhancing their interpersonal, communication skills. Moreover, they called for crash courses throughout their student training as well as lifelong learning activities aimed at updating knowledge and refurbishing skills to promote vaccine confidence. A study of immunization-related content in Canadian health science departments showcased a wide degree of variation within and across pharmacy, medical, and nursing departments, with the later devoting more time to respective curricula [6]. In addition, all studied curricula devoted less time to clinical skills, a feature similar to the issues raised by our participants. Adequate training prepares students for the challenge of communicating with hesitant patients, provides knowledge, and increases awareness and willingness among future health professionals to endorse vaccinations [18]. Ultimately, hesitancy is often linked with lack of trust in public institutions and pharmaceutical companies [7]. Regaining trust in the authorities is crucial in increasing vaccine confidence and proposed measures should target the public and health professionals alike, as low trust has been independently associated with vaccine hesitancy among health professionals as well [36,51]. As trust is gained through close and strong relationships between patients and health providers [7], informed and appropriately trained health providers are to extend patients’ confidence in ‘arbitrary’ actors such as health authorities and drug companies and persuade them to get vaccinated.

### 4.3. Strengths and Limitations of the Study

To the best of the authors’ knowledge, this is the first study in Greece, and among few studies internationally, to record undergraduate health sciences students’ placement on the vaccine acceptance continuum, as well as their perceptions on how to increase vaccine confidence. Moreover, it is the first study internationally to investigate the correlation of the relevant perceptions and practices with the God Locus of Health Control. It was the first study to be conducted in Greece shortly after the declaration of the end of the pandemic, allowing it to capture the possible effect of the universal vaccination against COVID-19 that preceded it. High response rates within university departments are also worth noting.

However, some limitations must be considered when interpreting the study results. Data on vaccination coverage were self-reported by participants, therefore potential errors in information recall may have led to underestimated or overestimated vaccination rates. As adults may not be able to accurately recall vaccinations they received as teenagers, HPV uptake levels may have been underestimated. However, the use of self-reported, questionnaire-based data appears to be an acceptable method in similar studies investigating vaccine coverage [23,67]. Moreover, another limitation of our study is the inclusion of only third-year students. As the third-year curriculum in both schools includes the commencement of clinical practice, we aimed at documenting the stances, knowledge and needs of health science students immediately before starting to face patients. Future research efforts could underscore attitudes and needs of students from different years of studies as well as recent graduates. As participation was voluntary, it is possible that vaccine advocates were more likely to take part in our study. Yet high response rates in both departments allow the study results to properly reflect health science students’ attitudes, beliefs, and practices. Ultimately, the 10 undergraduate students who participated in the pre-test of the study tool were invited to take part in the study. Given that no alterations in the study tool were performed, according to participants’ feedback, the latter may have been better equipped to respond than the rest of their counterparts [68]. Yet those students were informed about the nature and the scope of their participation in the pre-test stage, which was solely to provide feedback and indicate comprehension of the items of our study tool. Moreover, as the pre-test sample of students represents < 2.5% of the total participants, we do not consider the study results to have significantly skewed by the pretesting effect.

## 5. Conclusions

A quarter of medical and biomedical science students were in favor of all adult vaccinations, while the rest voiced some degree of hesitancy. Although participants’ stances towards adult vaccines were affirmative, vaccination rates against studied pathogens were suboptimal. Students believing in the value of adult vaccination and aware of national guidelines were more likely to have undertaken vaccination as adults, whereas the notion that God is in control of individual’s health only affected COVID-19 vaccine uptake. Participants, especially those trusting the value, safety, and knowledge of adult immunization, considered vaccination to be of pivotal importance as health science students and health professionals. Two-thirds of future health professionals will urge their patients to get vaccinated. Being a medical student, of excellent health status, and having a positive viewpoint over the value of immunization and fewer safety concerns were significant predictors in vaccine endorsement. Yet our study underscores moderate knowledge and confidence to address hesitancy among health science students. Analysis of qualitative data underpinned the importance of increasing vaccine health literacy, instilling trust in authorities, and adopting patient-centered approaches during hesitancy-related patient–provider conversations in addressing vaccine hesitancy. Reforms in undergraduate curricula and providing lifelong training support is pivotal in enhancing competence, strengthening vaccine confidence among future health providers, and promoting vaccination in the post-COVID-19 era.

## Figures and Tables

**Table 1:** Sociodemographic characteristics and health status of study participants. Percentage of valid responses and the absolute number are indicated.

Sample Characteristics	Medical Students *n*/N (%)	Biomedical Science Students *n*/N (%)
**Gender** Men Women	112/279 (40.1%) 167/279 (59.9%)	42/148 (28.4%) 106/148 (71.6%)
**Smoking**	29/281 (10.3%)	23/149 (15.4%)
**Chronic health condition**	35/280 (12.5%)	13/148 (8.8%)
**Health status (Self-assessed)** Moderate Good Very good Excellent	10/280 (3.6%) 39/280 (13.9%) 166/280 (59.3%) 65/280 (23.2%)	7/149 (4.7%) 39/149 (26.2%) 85/149 (57%) 18/149 (12.1%)

**Table 2:** Participant responses on the God Locus of Health Control (GLHC) scale per item (mean, SD). Percentage of valid responses and the absolute number after combining those falling into negative and positive ends of the scale are indicated.

	Medical Students			Biomedical Science Students		
	Mean (SD)	‘Strongly Disagree’/ ‘Moderately Disagree’ *n*/N (%)	‘Strongly Agree’/ ‘Moderately Agree’ *n*/N (%)	Mean (SD)	‘Strongly Disagree’/ ‘Moderately Disagree’ *n*/N (%)	‘Strongly Agree’/ ‘Moderately Agree’ *n*/N (%)
If my (health; condition) worsens, it is up to God to determine whether I will feel better again.	1.99 (1.18)	191/274 (69.7%)	8/274 (2.9%)	2.11 (1.31)	90/148 (60.8%)	7/148 (4.7%)
Most things that affect my (health; condition) happen because of God.	1.82 (1.11)	203/274 (74.1%)	6/274 (2.2%)	1.88 (1.06)	98/148 (66.2%)	1/148 (0.7%)
God is directly responsible for my (health; condition) getting better or worse.	1.92 (1.18)	193/274 (70.4%)	5/274 (1.8%)	1.97 (1.16)	92/148 (62.2%)	3/148 (2%)
Whatever happens to my (health; condition) is God’s will.	2 (1.32)	190/274 (69.3%)	17/274 (6.2%)	2.18 (1.37)	86/148 (58.1%)	9/148 (6.1%)
Whether or not my (health; condition) improves is up to God.	1.98 (1.23)	191/274 (69.7%)	8/274 (2.9%)	2.05 (1.21)	90/148 (60.8%)	2/148 (1.4%)
God is in control of my (health; condition).	1.87 (1.21)	197/274 (71.9%)	9/274 (3.3%)	2.01 (1.28)	96/148 (64.9%)	6/148 (4.1%)

**Table 3:** Attitudes towards adult vaccination according to the continuum of vaccine acceptance. Percentage of valid responses and the absolute number are indicated.

	Category in the Continuum of Vaccine Acceptance				
	In Favor of All Adult Vaccines *n*/N (%)	In Favor of Adult Vaccines, But Not Sure about All *n*/N (%)	In Favor of Some Adult Vaccines, But Delay or Refuse Some *n*/N (%)	Against Adult Vaccines, But Not Sure about All *n*/N (%)	Against All Adult Vaccines *n*/N (%)
**Medical students**	77/280 (27.5%)	184/280 (65.7%)	19/280 (6.8%)	-	-
**Biomedical science students**	32/149 (21.5%)	96/149 (64.4%)	18/149 (12.1%)	3/149 (2%)	-

**Table 4:** Participants’ Attitude Towards Adult Vaccination (ATAVAC) and God Locus of Health Control (GLHC) scores (mean, SD) according to standpoints towards adult vaccination.

Mean Score per Category	Category in the Continuum of Vaccine Acceptance									
	In Favor of All Adult Vaccines		In Favor of Adult Vaccines, But Not Sure about All		In Favor of Some Adult Vaccines, But Delay or Refuse Some		Against Adult Vaccines, But Not Sure about All		Against All Adult Vaccines	
	M *	B **	M *	B **	M *	B **	M *	B **	M *	B *
**ATAVAC scale**	5.41 (0.36)	5.31 (0.43)	5.04 (0.51)	4.78 (0.46)	4.64 (0.57)	4.42 (0.49)	-	4.24 (1.11)	-	-
Value of adult vaccination	5.55 (0.35)	5.39 (0.44)	5.17 (0.50)	4.92 (0.49)	4.68 (0.57)	4.62 (0.52)	-	4.24 (1.43)	-	-
Safety concerns	4.94 (0.98)	5.02 (1.00)	4.2 (1.20)	3.91 (1.12)	3.79 (1.27)	3.03 (1.40)	-	3.17 (1.76)	-	-
Perceived barriers	5.40 (0.68)	5.33 (0.79)	5.42 (0.65)	5.14 (0.68)	5.34 (0.53)	5.11 (0.78)	-	5.33 (0.76)	-	-
**GLHC**	10.2 (6.69)	9.53 (6.66)	12.1 (6.98)	12.4 (6.66)	11.9 (6.01)	16.2 (7.91)	-	7 (1.41)	-	-

* Medical students, ** Biomedical Science students.

**Table 5:** Attitude Towards Adult Vaccination (ATAVAC) and vaccine-related beliefs per item (mean, SD). Percentage of valid responses and the absolute number after combining those falling into negative and positive ends of the scale are indicated.

	Medical Students			Biomedical Science Students		
ATAVAC Items	Mean (SD)	‘Agree’/‘Strongly Agree’ *n*/N (%)	‘Disagree’/‘Strongly Disagree’ *n*/N (%)	Mean (SD)	‘Agree’/‘Strongly Agree’ *n*/N (%)	‘Disagree’/‘Strongly Disagree’ *n*/N (%)
I fear the immediate complications of a vaccine (such as allergic reactions)	4.43 (1.28)	26/281 (9.3%)	160/281 (56.9%)	4.06 (1.31)	19/149 (12.8%)	68/149 (45.6%)
I fear the potential impact of vaccines on my health in the future	4.33 (1.30)	27/281 (9.6%)	151/281 (53.7%)	3.99 (1.41)	28/149 (18.8%)	62/149 (41.6%)
I believe in the value of vaccination	5.67 (0.64)	270/281 (96%)	3/281 (1.1%)	5.43 (0.76)	136/149 (91.3%)	1/149 (0.7%)
It is difficult for me to access the doctor for vaccination (I cannot find an appointment, or the office is too far away or there is no transportation, etc.)	5.48 (0.74)	1/281 (0.4%)	259/281 (92.2%)	5.40 (0.71)	0/149 (0%)	138/149 (92.6%)
I believe that vaccines are necessary for adults	5.38 (0.75)	251/281 (89.3%)	0/281 (0%)	5.08 (0.84)	115/149 (77.2%)	1/149 (0.7%)
I believe that the benefits of vaccination outweigh the potential risks	5.46 (0.72)	252/281 (89.7%)	1/281 (0.4%)	5.07 (0.81)	114/149 (76.5%)	0/149 (0%)
I think if I get ill, I will get more antibodies (better body auto-defense) than if I just get a vaccination	4.04 (1.35)	41/281 (14.6%)	126/281 (44.8%)	3.66 (1.29)	35/149 (23.5%)	47/149 (31.5%)
I believe that vaccines are very effective in protecting me from getting a disease	4.74 (1.02)	201/281 (71.5%)	13/281 (4.6%)	4.57 (1.00)	89/149 (59.7%)	8/149 (5.4%)
I haven’t had a vaccine as an adult so far, so I don’t need it	5.76 (0.57)	1/281 (0.4%)	273/281 (97.2%)	5.60 (0.69)	1/149 (0.7%)	141/149 (94.6%)
I believe that vaccines should only be given to children	5.63 (0.66)	1/281 (0.4%)	267/281 (95%)	5.38 (0.61)	0/149 (0%)	139/149 (93.3%)
I have financial difficulty in paying for a visit to a doctor or I can’t afford the transportation costs to the office to have the vaccines I need.	5.33 (0.86)	5/281 (1.8%)	251/281 (89.3%)	4.95 (0.95)	2/149 (1.3%)	112/149 (75.2%)
**Additional items**						
I am afraid of needles; hence I avoid vaccines	5.78 (0.57)	1/281 (0.4%)	272/281 (96.8%)	5.60 (0.63)	0/149 (0%)	139/149 (93.3%)
I know people who got a vaccination that caused them a health issue	3.79 (1.59)	79/281 (28.1%)	114/281 (40.6%)	3.69 (1.53)	41/149 (25.7%)	56/149 (37.6%)
I believe that vaccines are another way for pharmaceutical companies to make money	4.32 (1.29)	28/281 (10%)	143/281 (50.9%)	3.91 (1.37)	20/149 (13.4%)	57/149 (38.3%)

**Table 6:** Perceptions on health professionals’ role in adult immunization and readiness to address hesitancy, rated on a 10-point Likert scale (from 1 = ‘not at all’ to 10 = ‘extremely important’).

	Medical Students (*n* = 279)	Biomedical Science Students (*n* = 149)
	Mean (SD)	
How important do you think it is for health sciences students to be vaccinated according to the National Adult Immunization Program?	8.99 (1.35) *	8.30 (1.70) *
How important do you consider vaccination according to the National Adult Immunization Program to be for those working as health professionals?	9.42 (0.97) *	8.99 (1.34) *
How informed do you feel so as to motivate a close acquaintance of yours to get vaccinated according to the National Adult Immunization Program?	6.90 (1.94)	6.60 (1.92)
How confident do you feel you will address vaccine-related hesitancy in people you will try to persuade?	6.91 (1.83)	6.78 (1.77)

* *p* < 0.001.

**Table 8:** Self-reported vaccination status of study participants. Percentage of valid responses and the absolute number are indicated.

	Total (*n* = 430)—*n*/N (%)	Medical Students (*n* = 281)—*n*/N (%)	Biomedical Science Students (*n* = 149)—*n*/N (%)
**Vaccination as an adult** Yes No Can’t Recall	381/429 (88.8%) 39/429 (9.1%) 9/429 (2.1%)	254/281 (90.4%) 21/281 (7.5%) 6/281 (2.1%)	127/148 (85.8%) 18/148 (12.2%) 3/148 (2%)
**Influenza ^1^** Yes No	79/428 (18.5%) 349/428 (81.5%)	57/279 (20.4%) 222/279 (79.6%)	22/149 (14.8%) 127/149 (85.2%)
**Tetanus, Dipthteria, Pertussis ^2^** Yes No Can’t Recall	129/429 (30.1%) 242/429 (56.4%) 58/429 (13.5%)	84/280 (30%) 161/280 (57.5%) 35/280 (12.5%)	45/149 (30.2%) 81/149 (54.4%) 23/149 (15.4%)
**COVID-19 ^3^** Yes *Full* *Partial* No	400/429 (93.2%) 377/396 (95.2%) 19/396 (4.8%) 29/429 (6.8%)	270/280 (96.4%) 257/266 (96.6%) 9/266 (3.4%) 10/280 (3.6%)	130/149 (87.2%) 120/130 (92.3%) 10/130 (7.7%) 19/149 (12.8%)
**HPV ^4^** Yes *Men* *Women* No *Men* *Women* Can’t Recall *Men* *Women*	226/429 (52.7%) 28/153 (18.3%) 198/273 (72.5%) 120/429 (28%) 72/153 (47.1%) 45/273 (16.5%) 83/429 (19.3%) 53/153 (34.7%) 30/273 (11%)	152/280 (54.3%) 23/111 (20.7%) 129/167 (75.4%) 79/280 (28.2%) 53/111 (47.7%) 24/167 (14.4%) 49/280 (17.5%) 35/111 (31.5%) 14/167 (8.4%)	74/149 (49.7%) 5/42 (11.9%) 69/106 (65.1%) 41/149 (27.5%) 19/42 (45.2%) 21/106 (19.8%) 34/149 (22.8%) 18/42 (42.9%) 16/106 (15.1%)

^1^ One dose during the 2022–2023 season recommended for health students as a high-risk group, ^2^ One dose for those 18–25 years old and/or a booster dose every 10 years, ^3^ COVID-19: Partial vaccination (1 dose); Full vaccination (≥2 doses), ^4^ Human papillomavirus: affirmative for underage (9–18 years old) or delayed vaccination (18–26 years old).

**Table 9:** Factors associated with vaccine uptake *.

Variables	Vaccinated as an Adult		Influenza		Tetanus, Diphtheria, Pertussis		COVID-19 ^1^		HPV ^1^	
	aOR ^2^ (95% CI)	*p*- Value	aOR ^2^ (95% CI)	*p*- Value	aOR ^2^ (95% CI)	*p*- Value	aOR ^2^ (95% CI)	*p*- Value	aOR ^2^ (95% CI)	*p*- Value
**Sociodemographic** **factors**										
Medical vs. Biomedical science student	1.12 (0.57–2.17)	0.746	1.06 (0.59–1.89)	0.843	-	-	2.65 (1.06–6.61)	0.037	-	-
Females vs. males	-	-	-	-	0.78 (0.50–1.24)	0.294	-	-	19.45 (10.06–37.63)	<0.001
**Health status factors**										
Chronic condition	3.07 (0.67–14.05)	0.147	2.86 (1.46–5.58)	0.002	-	-	-	-	-	-
**ATAVAC subscales ^3^**										
Value of adult vaccination	2.18 (1.09–4.35)	0.027	1.31 (0.67–2.58)	0.432	0.56 (0.36–0.88)	0.011	4.80 (1.86–12.41)	0.001	2.66 (1.41–5.01)	0.002
Safety concerns	1.12 (0.82–1.52)	0.473	1.08 (0.84–1.39)	0.564	-	-	1.71 (1.12–2.60)	0.013	1.48 (1.12–1.95)	0.006
Perceived barriers	-	-	-	-	-	-	-	-	1.40 (0.91–2.14)	0.125
**GLHC ^4^**	-	-	-	-	1.02 (0.99–1.05)	0.230	0.91 (0.85–0.97)	0.003	-	-
**Vaccination-related attitudes and beliefs**										
Not afraid of needles	0.95 (0.58–1.56)	0.832	2.04 (0.99–4.17)	0.050	-	-	-	-	1.05 (0.68–1.65)	0.818
Vaccines are not another way for drug companies to make money	1.08 (0.81–1.43)	0.609	1.15 (0.92–1.45)	0.218	-	-	0.95 (0.63–1.44)	0.803	0.90 (0.71–1.15)	0.403
In favor of all adult vaccines	0.89 (0.35–2.28)	0.807	1.31 (0.72–2.37)	0.378	-	-	-	-	-	-
Informed about adult vaccination guidelines	1.19 (1.02–1.39)	0.032	1.09 (0.93–1.27)	0.300	1.21 (1.06–1.39)	0.005	0.98 (0.77–1.25)	0.867	-	-

* Missing values represent variables with no statistically significant correlation in the univariable models, therefore not tested in the multivariable models. ^1^ Human papillomavirus, ^2^ Adjusted odds ratio, ^3^ Attitudes Towards Adult Vaccination, ^4^ God Health Locus of Control.

**Table 10:** Illustrative quotes from the open-ended question exploring the enablers in addressing vaccine hesitancy as future health professionals, allocated in overarching themes. Participants’ number and study department are presented for each quote.

Themes	Illustrative Quotes
Increased health literacy in the community	“Adequate information about the nature and effectiveness of vaccination could address vaccine-related stereotypes.” (P41, Medical student) “Awareness on the value of vaccination not only from the National Health System but also from other state actors, mainly the educational system.” (P136, Medical student) “Education. Provide comprehensible, understandable arguments in favor of vaccination. Children should learn the value of vaccination and related themes at a young age, so that they are fully informed in adulthood.” (P190, Medical student) “[Providing] information and awareness regarding public and personal health.” (P291, Biomedical science student)
Patient-centered approach towards users of health services	“Clear explanation of how vaccines work; persuasion is to be based on understanding.” (P97, Medical student) “Condemning any conspiracy thoughts that prompt patients to not vaccinate.” (P113, Medical students) “Avoiding misinformation, providing person-centred health.” (P116, Medical student) “Gentle approach of hesitant patients, combined with simple and easy-to-comprehend analysis of vaccine-induced mechanisms.” (P120, Medical student) “Presenting data that support the benefits of administering a vaccine for optimal health care delivery, provided that this is indeed reliable.” (P125, Medical student) “Bridging the gap with patient understanding.” (P239, Medical student) “Explaining how vaccines work, analyzing benefits, providing facts and statistics, whilst not undermining patient’s views or mock their fears.” (P341, Biomedical science student)
Health professionals’ training and education	“Having the necessary knowledge to help patients understand the importance of vaccination and to reassure them if they express hesitancy.” (P13, Medical student) “Training so that health professionals can analyze in a simple, understandable, and accurate way the benefits of vaccination, without omitting the side effects.” (P34, Medical student) “During our undergraduate as well as postgraduate studies, seminars and lectures regarding the appropriate, convincing and effective way of approaching vaccine skeptics should be organized.” (P144, Medical students) “Medical education should be improved, providing the opportunity for students to acquire soft skills such as communication.” (P252, Medical student) “Accurate and valid knowledge of the subject gives me confidence. Yet, in the long-run, I think that the patient can obtain a level of more trust and security [in me].” (P313, Biomedical science student) “[Gaining] more knowledge on the subject to be able to develop arguments and persuade [patients].” (P326, Biomedical science student)
Boosting trust in authorities and institutions	“Confidence in the National Vaccination Program and those involved, who are interested in citizens’ health and not in drug companies’ profits.” (P199, Medical student) “The development of relationships of trust between the health system and patients.” (P226, Medical student) “Establishment of vaccination centers in more areas.” (P276, Medical student) “[Vaccination] being optional and without repercussions. This approach seems less threatening and oppressive when social injustices occur against the unvaccinated.” (P316, Biomedical science student) “Debunking myths about financial interests of pharmaceutical companies, detailed and comprehensible information so that citizens reject conspiracy theories.” (P422, Biomedical science student)

**Table 7:** Factors associated with the importance of getting vaccinated as health sciences students, health professionals as well as vaccine endorsement *.

Variables	Importance of Getting Vaccinated as Health Sciences Students		Importance of Getting Vaccinated as Health Professionals		Vaccination Endorsement	
	aOR ^1^ (95% CI)	*p*- Value	aOR ^1^ (95% CI)	*p*- Value	aOR ^1^ (95% CI)	*p*- Value
**Sociodemographic** **factors**						
Medical vs. Biomedical science student	2.03 (1.18–3.47)	0.010	1.34 (0.71–2.56)	0.366	2.45 (1.33–4.48)	0.004
Females vs. males	2.48 (1.40–4.39)	0.002	-	-	-	-
**Health status factors**						
Vaccinated as adult	0.86 (0.38–1.98)	0.728	1.99 (0.85–4.67)	0.112	1.36 (0.60–3.09)	0.469
Chronic condition	1.48 (0.62–3.54)	0.381	-	-	1.96 (0.62–6.22)	0.254
Excellent health status	-	-	-	-	2.27 (1.14–4.52)	0.020
**ATAVAC subscales ^2^**						
Value of adult vaccination	3.43 (1.83–6.43)	<0.001	5.39 (2.55–11.40)	<0.001	3.26 (1.65–6.42)	<0.001
Safety concerns	1.38 (1.08–1.77)	0.011	1.46 (1.07–1.98)	0.016	1.36 (1.02–1.80)	0.037
Perceived barriers	1.20 (0.80–1.79)	0.382	0.73 (0.44–1.21)	0.222	1.24 (0.78–1.96)	0.361
**GLHC ^3^**	1.00 (0.96–1.04)	0.881	1.00 (0.95–1.05)	0.957	0.98 (0.93–1.02)	0.312
**Vaccination-related attitudes and beliefs**						
Not afraid of needles	1.19 (0.79–1.80)	0.403	-	-	0.93 (0.60–1.45)	0.758
Vaccines are not another way for drug companies to make money	1.15 (0.92–1.44)	0.226	1.11 (0.83–1.48)	0.485	1.11 (0.85–1.45)	0.460
In favor of all adult vaccines	1.49 (0.71–3.12)	0.291	1.13 (−0.37–3.38)	0.83	2.18 (0.76–6.30)	0.149
Informed about adult vaccination guidelines	1.38 (1.15–1.65)	<0.001	1.31 (1.07–1.59)	0.008	1.18 (0.98–1.43)	0.078
Confident to manage vaccine hesitancy	1.04 (0.86–1.26)	0.668	1.12 (0.91–1.39)	0.289	1.20 (0.97–1.47)	0.088

* Scoring ≥ 9 on a 10-point Likert scale was set as the cut-off point indicating importance of getting vaccinated as health sciences students and health professionals. Missing values represent variables with no statistically significant correlation in the univariable models, therefore not tested in the multivariable models. ^1^ Adjusted odds ratio, ^2^ Attitudes Towards Adult Vaccination, ^3^ God Locus of Health Control.

## Data Availability

The data presented in this study are available on request from the corresponding author.
